# Commentary: Asymmetric adult-onset asthma with periocular xanthogranuloma associated with IgG4-related disease: a case report

**DOI:** 10.3389/fsurg.2026.1787830

**Published:** 2026-04-22

**Authors:** Yue Cheng, Qianqian Mi, Dan Wang

**Affiliations:** 1 Changchun University of Chinese Medicine, Changchun, China; 2 Jilin Provincial People’s Hospital, Changchun, China

**Keywords:** AAPOX, adult-onset asthma, AIT, allergen-specific immunotherapy, AOXGD, igG4-RD, igG4-related disease, orbital xanthogranulomatous disease

## Introduction

We read with great interest the article recently published by Huang and Chen in Frontiers in Surgery, titled “Asymmetric adult-onset asthma with periocular xanthogranuloma (AAPOX) associated with IgG4-related disease: a case report” (1). The authors detailed a rare case of AAPOX characterized by significant asymmetric eyelid swelling. Notably, the patient had a documented history of asthma and allergen-specific immunotherapy (AIT). Furthermore, postoperative pathological analysis revealed a low ratio of IgG4-positive to IgG-positive plasma cells (<40%), a finding attributed to the underlying xanthogranulomatous background (1). This case not only expands the clinical spectrum of adult orbital xanthogranulomatous disease (AOXGD) but also provides a compelling clinical model for exploring the potential association between AIT and the pathogenesis of IgG4-related disease (IgG4-RD). Herein, we aim to discuss the diagnostic challenges associated with this condition and the therapeutic paradox—specifically the bidirectional immunomodulatory effects—of immunotherapy highlighted by this case.

## Diagnostic dilemmas: the histological challenge of AAPOX

The diagnosis of IgG4-RD is typically established based on the 2019 ACR/EULAR classification criteria, with histopathology serving as the cornerstone (2). Huang and Chen reported that the immunohistochemical analysis exhibited an IgG4/IgG plasma cell ratio of less than 40%. Consequently, this finding failed to meet the conventional pathological threshold for diagnosis (1). This finding underscores a critical controversy in IgG4-RD diagnosis: the applicability of universal histological cutoffs across all subtypes. Previous studies have demonstrated significant variability in the density of IgG4 + cell infiltration across different organs; consequently, strict adherence to the >40% ratio may reduce diagnostic sensitivity in certain fibrotic or sclerotic lesions (3). We postulate that this phenomenon is particularly prominent in the AAPOX subtype.

As a xanthogranulomatous disorder, AAPOX is pathologically characterized by a predominance of foam cells and Touton giant cells (4). This pronounced histiocytic reaction may result in the spatial dilution of IgG4-positive plasma cells, thereby interfering with the denominator during ratio calculation and leading to a “false-negative” score. Furthermore, although the significant asymmetry observed in this case is uncommon in classical AAPOX, bilateral lacrimal gland involvement was confirmed via imaging. These findings suggest that for orbital masses accompanied by a severe asthmatic background, the diagnosis of IgG4-RD should be considered flexibly. A comprehensive evaluation is essential even when the IgG4/IgG ratio is suboptimal or the clinical appearance is asymmetric. This process should involve careful evaluation of serological markers—such as the extremely high IgE (1,520 IU/mL) and the significantly elevated post-operative serum IgG4 (11.80 g/L) observed in this case—alongside systemic assessment to avoid misdiagnosis caused by rigid adherence to standard criteria (5). To address these diagnostic challenges, we recommend a multi-modal integration strategy. When the histological IgG4/IgG ratio is suboptimal, clinicians should prioritize the “clinical-radiological-serological” triad. Drawing from the clinical features of this case and the known pathophysiology of AAPOX, we suggest conceptually stratifying these indicators to guide clinical reasoning. Core indicators comprise the established anatomical and systemic hallmarks of IgG4-RD, specifically bilateral lacrimal gland/extraocular muscle enlargement on imaging and elevated serum IgG4. Auxiliary indicators reflect the distinct Th2-driven atopic background characteristic of the AAPOX phenotype, notably severe adult-onset asthma and markedly elevated serum IgE.

Given the rarity of AAPOX, establishing rigid numerical diagnostic criteria remains challenging without large-cohort validation. However, we propose that a “probable” diagnosis is highly justified when a suboptimal pathological result is counterbalanced by definitive core imaging features, particularly when accompanied by auxiliary atopic indicators. In such diagnostic dilemmas, the “probable” diagnosis should be pragmatically corroborated by an ex juvantibus (diagnostic-by-treatment) approach using a systemic corticosteroid trial. To enhance clinical utility, we recommend evaluating this therapeutic response across three qualitative dimensions: (1) Clinical: A discernible reduction in periocular swelling and xanthogranulomatous mass, alongside stabilization of asthmatic symptoms; (2) Radiological: A demonstrable regression of lacrimal gland or orbital mass volume on follow-up imaging; and (3) Serological: A downward trajectory of serum IgG4 and IgE levels that parallels clinical improvement.The comparative clinical and pathological nuances between typical AAPOX, classical orbital IgG4-RD, and the present case are summarized in Table 1.

**Table 1:** Comparison of clinical-pathological features between AAPOX and classical orbital IgG4-RD.

Features	Typical AAPOX	Classical Orbital IgG4-RD	Features of Huang & Chen's Case
Clinical Presentation	Bilateral, symmetric eyelid/orbital swelling; yellow plaques (xanthomas).	Unilateral or bilateral swelling; lacrimal gland enlargement common; no yellow plaques.	Asymmetric (Right > Left); xanthogranulomatous mass.
Systemic Association	Adult-onset asthma (severe); lymphadenopathy.	Multi-organ involvement (pancreas, retroperitoneum, etc.); history of allergy common.	Asthma; High IgE; High serum IgG4.
Serum IgG4	Variable; often elevated.	Elevated in >60–70% of cases (>135 mg/dL).	Significantly elevated (11.80 g/L post-operatively).
Histopathology	Xanthogranulomatous: Foam cells, Touton giant cells, necrotic foci.	Lymphoplasmacytic: Dense lymphocytes, IgG4 + plasma cells, storiform fibrosis.	Xanthogranulomatous (Foam cells) + Lympho-plasmacytic infiltration.
IgG4/IgG Tissue Ratio	Variable; often <40% (due to histiocytic dominance).	>40% is a key diagnostic criterion.	<40% (Did not meet strict diagnostic criteria).
Treatment Response	Corticosteroids (variable response), Surgery (debulking).	Corticosteroids (excellent response), Rituximab.	Surgery + Corticosteroids (Good response).

## The immunotherapy paradox: From protection to pathogenesis

The most thought-provoking aspect of this case is the patient's history of undergoing asthma desensitization therapy (AIT) for three months (1). The authors hypothesized that this treatment may have facilitated the production of IgG4. This hypothesis touches upon a fundamental paradox in immunology: how does the “protective” IgG4 induced by AIT transform into a “pathogenic” factor in IgG4-RD?

In the context of allergy treatment, AIT is designed to induce immune tolerance. Its mechanism relies on IL-10-driven class switching in B cells, which leads to the production of specific IgG4 antibodies that block IgE-mediated allergic reactions (6). However, within the pathophysiology of IgG4-RD, there is a complex mechanistic overlap between allergy and autoimmunity. Research has confirmed that patients with IgG4-RD widely exhibit a Th2-type immune bias, and IgG4 levels are positively correlated with IgE levels and eosinophil counts (7, 8). The patient in this case exhibited extremely high IgE levels (1,520 IU/mL), indicating a potent Th2 inflammatory environment.

Furthermore, in-depth mechanistic studies have revealed oligoclonal expansions of circulating plasmablasts in patients with active IgG4-RD, suggesting that the disease is driven by specific antigens (9). The transition from protective to pathogenic IgG4 responses likely involves a “two-hit” mechanism. First, in the context of specific genetic susceptibility or Th2 hyperresponsiveness, chronic antigen exposure during AIT may lead to dysregulated IgG4 production. Notably, T follicular helper 2 (Tfh2) cells, which are expanded in IgG4-RD and correlate with disease activity (10), play a critical role in this transition. These Tfh2 cells provide aberrant help to B cells, facilitating their differentiation into plasmablasts and enhancing IgG4 class switching through IL-4 and IL-21 production (11). While physiological IgG4 responses in AIT are typically polyclonal and allergen-specific, the pathogenic state in IgG4-RD is characterized by the emergence of oligoclonal expansions and extensive somatic hypermutation, suggesting selection by self-antigens or molecular mimicry (9). Specifically applying this to the present case, the patient's severe adult-onset asthma and extremely high baseline IgE level (1,520 IU/mL) perfectly exemplify this hyperresponsive Th2 state, allowing the 3-month AIT to act as a continuous trigger that breached the quantitative threshold of this first hit.

Second, the structural properties of IgG4 create a paradoxical pathogenic potential. While Fab-arm exchange typically produces functionally monovalent, bispecific antibodies that limit immune complex formation, under conditions of high antigen density found in fibro-inflammatory lesions, IgG4 can paradoxically activate the classical complement pathway (12). Furthermore, aberrant glycosylation patterns may influence IgG4's effector functions and disease severity (12). Recent experimental evidence further elucidates this second hit. A landmark study by Oskam et al. (2023) demonstrated that under conditions of critically high antibody concentrations and high antigen density, IgG4 is forced into a hexameric conformation due to spatial crowding, thereby efficiently activating the classical complement pathway (12). Additionally, recent glycomics research confirms that aberrant agalactosylation of IgG4 in autoimmune microenvironments strongly activates the lectin complement pathway (13). In the present case, the dense periocular xanthogranulomatous lesion—packed with histiocytes and foam cells—served as a high-density “antigen sink.” Accompanied by the profoundly elevated post-operative serum IgG4 (11.80 g/L, nearly nine times the conventional threshold), this unique local microenvironment provided the exact extreme spatial crowding required to overcome IgG4's inertness, triggering the rapid and asymmetric tissue destruction observed.

We hypothesize that in the context of specific genetic susceptibility or Th2 hyperresponsiveness, the IgG4 production artificially induced by AIT may become unregulated. Consequently, what begins as an “antigen-specific, protective” response may evolve into a pathogenic oligoclonal systemic fibro-inflammatory reaction. This “immunotherapy paradox” serves as a cautionary note to clinicians: in patients with high IgE levels undergoing AIT, the emergence of atypical tissue swelling or abnormally elevated serum IgG4 should raise suspicion regarding the risk of iatrogenic systemic IgG4-RD.

## Discussion

The approach of “surgical debulking combined with corticosteroid therapy,” as employed by Huang and Chen, demonstrated efficacy in this case (1). Surgery not only confirmed the diagnosis but also directly alleviated the mechanical ptosis caused by fibrosis and lipid deposition—conditions that are difficult to reverse rapidly with pharmacological intervention alone.

Given the adverse effects of corticosteroids observed in the patient and the propensity of IgG4-RD for recurrence, establishing a rigorous pharmacovigilance protocol is paramount. Long-term corticosteroid use necessitates multidisciplinary monitoring, including baseline and routine intraocular pressure (IOP) and slit-lamp evaluations to prevent ocular toxicities like glaucoma or subcapsular cataracts, alongside dual-energy x-ray absorptiometry (DEXA) scans and metabolic assessments to mitigate systemic risks (14). Consequently, future therapeutic strategies should consider the introduction of rituximab. By depleting B-cell precursors, rituximab has been shown to effectively control relapsing or refractory IgG4-RD and has demonstrated significant efficacy in orbital lesions accompanied by asthma (15). However, to prevent irreversible fibrotic relapses, the empirical dosing of rituximab should be guided by protocolized flow cytometry monitoring; sustaining CD19+ B-cell depletion (maintaining counts <10 cells/µL) is critical, and any repopulation above this threshold serves as an indication for pre-emptive re-treatment (16). In addition to B-cell depletion, conventional steroid-sparing agents such as mycophenolate mofetil or methotrexate remain important options for maintaining remission in refractory IgG4-RD (17).

Furthermore, emerging targeted therapies are providing more precise interventions. Inebilizumab, a humanized anti-CD19 monoclonal antibody, has recently been shown in a landmark phase 3 trial to significantly reduce the risk of flares in patients with IgG4-RD (18). Positioned as a first-line biologic disease-modifying antirheumatic drug (DMARD), inebilizumab should ideally be initiated concurrently with the acute corticosteroid tapering phase. For this specific patient, whose postoperative serum IgG4 remained alarmingly high (11.80 g/L), targeting CD19 is highly suitable as it more comprehensively depletes both B-cells and CD20-negative plasmablasts—the primary source of massive IgG4 production. Before initiation, rigorous screening for hepatitis B and tuberculosis, alongside longitudinal monitoring of serum immunoglobulins, is required to prevent severe hypogammaglobulinemia (18).

Given the potent Th2-type inflammatory background and highly elevated IgE levels in this AAPOX case, dupilumab—an IL-4 and IL-13 receptor antagonist—could serve as a personalized therapeutic option, as preliminary evidence suggests its efficacy in controlling both orbital xanthogranulomatous inflammation and underlying severe asthma (19). Ideally introduced early as a steroid-sparing agent, dupilumab precisely matches this patient's phenotype by directly blocking the upstream Tfh2-driven IgG4 class switching triggered by prior AIT. Nevertheless, clinical application requires strict pharmacovigilance for dupilumab-associated ocular surface disease (DAOSD). Particularly for a patient recovering from periocular surgery, baseline dry eye screening and prophylactic administration of preservative-free artificial tears are essential to distinguish DAOSD-induced conjunctivitis from an IgG4-RD orbital flare (20).

In conclusion, the case reported by Huang and Chen underscores the necessity of transcending histological ratio limitations in the diagnosis of AAPOX. Moreover, it provides early clinical evidence suggesting a potential causal link between asthma desensitization therapy and the onset of IgG4-RD.
